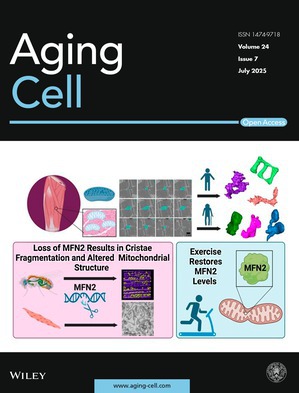# Featured Cover

**DOI:** 10.1111/acel.70170

**Published:** 2025-07-16

**Authors:** Estevão Scudese, Andrea G. Marshall, Zer Vue, Vernat Exil, Benjamin I. Rodriguez, Mert Demirci, Larry Vang, Edgar Garza López, Kit Neikirk, Bryanna Shao, Han Le, Dominique Stephens, Duane D. Hall, Rahmati Rostami, Taylor Rodman, Kinuthia Kabugi, Jian‐qiang Shao, Margaret Mungai, Salma T. AshShareef, Innes Hicsasmaz, Sasha Manus, Celestine N. Wanjalla, Aaron Whiteside, Revathi Dasari, Clintoria R. Williams, Steven M. Damo, Jennifer A. Gaddy, Brian Glancy, Estélio Henrique Martin Dantas, André Kinder, Ashlesha Kadam, Dhanendra Tomar, Fabiana Scartoni, Matheus Baffi, Melanie R. McReynolds, Mark A. Phillips, Anthonya Cooper, Sandra A. Murray, Anita M. Quintana, Nelson Wandira, Okwute M. Ochayi, Magdalene Ameka, Annet Kirabo, Sepiso K. Masenga, Chanel Harris, Ashton Oliver, Pamela Martin, Amadou Gaye, Olga Korolkova, Vineeta Sharma, Bret C. Mobley, Prasanna Katti, Antentor Hinton

## Abstract

Cover legend: The cover image is based on the article *3D Mitochondrial Structure in Aging Human Skeletal Muscle: Insights Into MFN‐2‐Mediated Changes* by Estevão Scudese et al., https://doi.org/10.1111/acel.70054